# Effectiveness of a complex, pre-conception intervention to reduce the risk of diabetes by reducing adiposity in young adults in Malaysia: The Jom Mama project – A randomised controlled trial

**DOI:** 10.7189/jogh.12.04053

**Published:** 2022-08-17

**Authors:** Ainul NM Hanafiah, Jens Aagaard-Hansen, Julius CH Cheah, Shane A Norris, Zulkarnain BA Karim, Jutta KH Skau, Zainudin M Ali, Regien Biesma, Priya Matzen, Lokman H Sulaiman, Mark Hanson

**Affiliations:** 1 Institute for Health Systems Research, National Institutes of Health, Ministry of Health Malaysia, Selangor, Malaysia; 2 Health Promotion Research, Steno Diabetes Center Copenhagen, Herlev, Denmark; 3 SA MRC Developmental Pathways for Health Research Unit, Department of Paediatrics, School of Clinical Medicine, Faculty of Health Sciences, University of the Witwatersrand, Johannesburg, South Africa; 4 School of Medicine and Health Sciences, Monash University Malaysia, Selangor, Malaysia; 5 Negeri Sembilan State Health Department, Seremban, Negeri Sembilan, Malaysia; 6 Universiti Teknologi Malaysia Kuala Lumpur, Kuala Lumpur, Malaysia; 7 Global Health Unit, Department of Health Sciences, Faculty of Medical Sciences, University of Groningen, Groningen, the Netherlands; 8 Human Development and Health, Faculty of Medicine, University of Southampton, Southampton, UK; 9 Office of the Deputy Director General of Health (Public Health), Ministry of Health, Putrajaya, Malaysia; 10 Institute for Research, Development and Innovation, International Medical University Malaysia, Kuala Lumpur, Malaysia; 11 Institute of Developmental Sciences, British Heart Foundation Professor of Cardiovascular Sciences, Faculty of Medicine and NIHR Biomedical Research Centre, University of Southampton, Southampton, UK

## Abstract

**Background:**

Pre-conception interventions have the potential to lower non-communicable disease risk in prospective parents and reduce transmission of risk factors such as obesity to the next generation. The Jom Mama project in Malaysia investigated the effectiveness of a combined behaviour change communication and e-health intervention in young married couples prior to first pregnancy. This paper reports the evaluation of the effectiveness of this trial.

**Methods:**

Jom Mama was a non-blinded, randomised controlled trial (RCT) conducted in Seremban, Malaysia, over a period of 33 weeks, covering six contact points between trained community health workers and newly married couples before the conception of a first child. Out of 2075 eligible nulliparous women, 549 participated and 305 completed the intervention, with 145 women in the intervention and 160 in the control group. The intervention group received a complex behavioural change intervention, combining behaviour change communication provided by community health promoters and access to a habit formation mobile application, while the control group received the standard care provided by public health clinics in Malaysia. The primary outcome was a change in the woman’s waist circumference. Secondary outcomes were anthropometric and metabolic measures, dietary intake (Food Frequency Questionnaire, FFQ), physical activity (International Physical Activity Questionnaire, IPAQ) and mental health (Depression Anxiety Stress Scale, DASS 21). An extensive process evaluation was conducted alongside the trial in order to aid the interpretation of the main findings.

**Results:**

There were no significant differences of change in the woman’s waist circumference between intervention and control groups at the start and end of the intervention. While the weight, waist circumference and Body Mass Index (BMI) of women in both groups increased, there was a significantly lower increase in the intervention vs the control group over the period of the trial among women who are obese (0.1 kg vs 1.7 kg; *P* = 0.023, in the intervention and control group respectively). In terms of BMI, the obese intervention subgroup showed a slight reduction (0.01) compared to the obese control subgroup whose BMI increased by 0.7 (*P* = 0.015). There were no changes in the other secondary outcomes.

**Conclusions:**

The Jom Mama pre-conception intervention did not lead to a reduction in waist circumference or significant changes in other secondary outcomes over the eight months prior to conception. However, there was a significantly smaller weight gain in the intervention vs the control group, predominantly in women with pre-existing obesity.

Non-communicable diseases (NCDs) account for over 70% of deaths globally each year, especially in low- and middle-income countries undergoing socioeconomic transitions to Western lifestyles and diets [1]. Diabetes mellitus (DM) is one of the most common, but also least recognised NCDs worldwide. Today, 387 million people are living with diabetes, of whom 46.3% remain undiagnosed. The prevalence of DM is expected to increase to 53% by 2035 [1]. DM is an important public health concern in Malaysia, a middle-income country which has witnessed an increase in DM prevalence among adults aged 18 years and above from 11.2% in 2011 to 18.3% in 2019, with a higher prevalence of known diabetes among women (9.8%) vs men (9.0%) [2]. Parallel to this is an equally high prevalence of gestational diabetes, affecting close to one-third (27.9%) of Malaysian women. Similar trends have also been shown for hypertension and obesity [3].

Epidemiological data and findings from basic science research suggest that intervening to stop negative behaviours affecting both men’s and women’s weight, nutrition, physical activity, and health behaviours, such as smoking, alcohol consumption and stress, can substantially improve health indicators [4]. From the life-course perspective, the pre-conception period offers the opportunity to reduce NCD risk in both parents and children [5,6]. However, motivating young people to change behaviour at that point in life is challenging, requiring more than just the provision of information and opportunities [4].

Consequently, the Malaysian complex pre-conception intervention, Jom Mama (translated as “Come on, mother” in Bahasa Malaysia), was designed to “reduce adiposity” in young, recently married, nulliparous women and their partners. Malaysia was chosen as a site for a pre-conception intervention trial because of its well-established public health system based around regional health clinics serving specific populations. Additionally, couples in Malaysia planning to marry routinely seek health screening, especially for Human Immunodeficiency Virus (HIV) status, prior to obtaining a marriage licence. Finally, the average age for marriage is 26 years for women and 28 years for men [7], and many couples are planning to start a family soon after marriage.

The Jom Mama project was initiated in 2012 and conducted in three phases: 1) a formative phase to understand the problem, the lifestyle of young couples, and identify barriers to and facilitators of health [8-11]; 2) development of an intervention package based on the results from phase 1; and 3) phase for evaluating the intervention package through an RCT [12]. We tested the hypothesis that a complex lifestyle intervention delivered to women and their spouses for 8 months prior to pregnancy would lead to a decrease in the woman’s waist circumference.

## METHODS

### Design and sampling

The trial (conducted from November 2015 to December 2017) was designed as a non-blinded RCT to assess the effectiveness of a complex behaviour change intervention in improving the overall health of young women prior to their first pregnancy. All women planning to get married in the district of Seremban, in the state of Negeri Sembilan, Malaysia were targeted for participation. The inclusion criteria for participation were: 1) female and between 20-39 years of age; 2) nulliparous; 3) not pregnant at the time of signing the informed consent form; 4) owning a smartphone, with either an Android operating system version 4.1 and above or an iOS operating system 7.0 and above; and 5) having internet access. Women undergoing treatment for type 1 or 2 diabetes mellitus or not residing in the district of Seremban were excluded.

Seremban district has a similar ethnic composition and diabetes prevalence to the national average [9]. Five health clinics in Seremban were identified as data collection sites. A group of primary health care nurses was appointed as data collectors and they received specialised training in data collection. They were not the same nurses who were trained to carry out behaviour change communication (BCC), as they were part of the intervention team. The intervention follow-up period was approximately 33 weeks. The trial was designed following the Standard Protocol Items: Recommendations for Interventional Trials (SPIRIT) 2013 statement [12].

Women were randomised into the intervention and control arms at the baseline measurement visit with a 1:1 allocation ratio, using computer-generated random allocation sequences with block sizes of six subjects. Each clinic was provided with a sufficient number of identification (ID) numbers with randomisation codes. When a subject was enrolled in the trial, the study nurse assigned the lowest available ID number to the subject from the list of ID numbers. The Institute for Health System Research (IHSR) of the Malaysian Ministry of Health (the main implementation partner) prepared the randomisation lists which were then distributed to the five designated primary health clinics in Seremban. A copy was maintained by IHSR. Subjects in the control arm received standard care as provided by public health clinics in Malaysia, which did not involve contact with a community health promoter (CHP) or access to the Jom Mama app. Control subjects received one phone call from a research officer towards the end of the trial period to remind them of their endpoint visit. This paper’s authors were blinded to the randomisation codes.

### Outcome measures

The primary outcome of the RCT was a change in the woman’s WC from baseline to after 33 weeks. Secondary outcomes were differences between intervention and control groups in any change from baseline to after 33 weeks or between the groups at the endpoint in the following variables: weight; body mass index (BMI) following the WHO Asian population cut-off of underweight (<18.5 kg/m^2^), normal (18.5-22.9 kg/m^2^), overweight (23.0-27.4 kg/m^2^), obese (≥27.5 kg/m^2^); waist-to-height ratio; waist-to-hip ratio; glycated haemoglobin A1c (HbA1c); fasting lipid profile (total cholesterol, low-density lipoprotein cholesterol, high-density lipoprotein cholesterol, and triglycerides); systolic and diastolic blood pressure; diet as measured by a Food Frequency Questionnaire (FFQ) adapted from a locally validated version [13]; physical activity and sedentary behaviour as measured by the International Physical Activity Questionnaire (IPAQ); and mental health as measured by the Depression Anxiety and Stress Scale 21-item (DASS-21) [12]. Participants answered the FFQ, IPAQ, and DASS-21 self-reported questionnaire prior to having their anthropometric measurements and blood samples taken by the study nurse. Measurements for waist circumference, hip circumference, weight, height, and blood pressure followed standardised procedures based on World Health Organisation (WHO) STEPS surveillance. For lipid profile and HbA1c, a 10ml fasting (minimum 9 hours) blood sample was taken by the study nurse at the study sites. These procedures followed the trial protocol outlined in Skau et al. [12].

### Sample size

The calculation was based on the primary outcome of achieving a 2 cm reduction in change in waist circumference (WC) between intervention and control groups at intervention completion (33 weeks). Assuming a 5% level of significance, a statistical power of 90% and a standard deviation (SD) of WC of 5 cm, and using a two-tailed *t* test, 132 women per arm were required. Assuming a 20% attrition rate and an exclusion of 20% of participants due to them becoming pregnant before the trial’s completion period, a total of 660 women would be required to ensure a minimum of 264 subjects split between the intervention and control arms.

### Intervention components

The intervention aimed to encourage healthy lifestyle changes in young married couples through a combined behaviour change communication method implemented by community health promoters (CHPs) during six contact points (CPs), along with continuous access to a tailored mobile application. The 48 CHPs were recruited from within the public health care system and comprised 37 community health nurses, 10 staff nurses, and one nursing sister. They were given a four-day training course prior to the intervention, which introduced them to the national guidelines for nutrition and physical activity, as well as how to carry out BCC and use the Jom app. The course curriculum was developed by local communication experts through a series of consultations with multiple stakeholders and assessments of trainees’ prior levels of knowledge and skills. The course participants were given a handbook explaining the course content and the outcomes were evaluated using a pre-post design. A system was established to provide “peer support” for the CHPs during the intervention. The Jom app was developed specifically for the Jom Mama study by an external commercial software developer (Cognizant Technology Solutions) and included a function which provided lifestyle challenges that the young couples could select. The challenges were focused either on healthy food or physical activity. The app also provided a resource section where the couples could access information on healthy lifestyles. Physical activity challenge examples included brisk walking for 15 minutes, using the stairs instead of the elevator, and doing planks; healthy food challenges included avoiding oily food, drinking plain water/non-sweetened beverages and avoiding soft drinks [12]. The intervention included a total of six CPs with the CHP; three face-to-face (CPs 1, 2, and 5) at a site of their choice and three via phone (CPs 3, 4, and 6) over a period of about 33 weeks [12].

### Control components

The control group received standard care which did not include contact with a CHP or access to the E-health platform. By eight months after the baseline visit, the control couples would receive a call from a research officer reminding them of their endpoint visit.

### Statistical analysis

Based on the primary outcome, the null hypothesis was that there would be no difference between the intervention and the control group in any change in WC over the period of the trial. The secondary outcomes, physical activity, and sedentary behaviour (IPAQ) were analysed using an independent sample *t* test between the intervention and control groups. As all the variables for diet (FFQ) and mental health (DASS-21) were ordinal, the independence between intervention and control groups was tested with the χ^2^ test. We categorised DASS scores as normal, moderate, and severe, in accordance with the scores for depression (0-4 = normal, 5-10 = moderate, ≥11 = severe), anxiety (0-3 = normal, 4-7 = moderate, ≥8 severe), and stress (0-7 = normal, 8-12 = moderate, ≥13 = severe). Bootstrapping was used to control for key sociodemographic factors in the primary and secondary analysis. The data analyses were conducted in IBM SPSS version 23.

### Research ethics

The study protocol version 4.0 dated August 12, 2015, received ethical and governance approvals by the Medical Research and Ethics Committee of the Ministry of Health, Malaysia (protocol number NMRR-14-904-21963) on September 21, 2015. The study was performed in accordance with the Declaration of Helsinki [14]. All subjects provided written informed consent prior to participation in the trial. All participation was voluntary and the subjects could elect to withdraw from the trial at any time. Anonymity and confidentiality of participants were assured. The trial was registered at clinicaltrials.gov (identifier: NCT02617693) on November 30, 2015.

## RESULTS

### Study population characteristics

**Figure 1:**
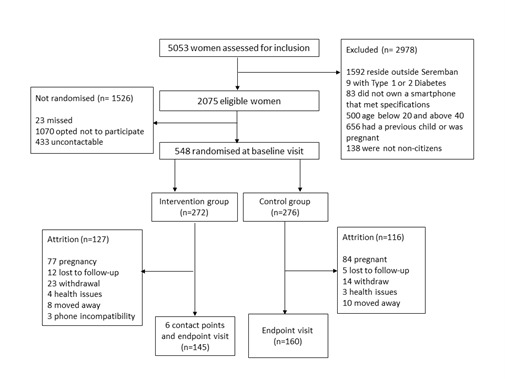
Jom Mama consort diagram illustrating the number of participants approached, eligible and recruited during the trial as well as the attrition.

Of the 5053 women approached for inclusion, 2075 women were found eligible, and out of these 548 women were randomised into either the intervention or control group (**Figure 1**) (response rate of 26.4%). Of these women, 305 (55.6%) completed the study. The main reason for loss to follow-up was pregnancy occurring during the study period.

Randomisation resulted in a near equivalence in baseline variables (**Table 1**), except that the mean age of women was higher in the intervention compared to the control group (29.1 years vs 27.9 years; *P* = 0.008).

**Table 1:** Baseline sociodemographic characteristics and values of anthropometric measures of study participants in intervention and control (standard care) groups of trial

Characteristics	Intervention n = 145)	Control (n = 160)	*P*-values
Age, years, mean (SD)	29.1 (4.3)	27.4 (4.1)	0.008
Waist circumference, cm, mean (SD)	81.4 (13.6)	80.7 (13.5)	0.640
Waist circumference above 80 cm, n (%)	69 (52.4)	77 (48.1)	0.928
Weight, kg, mean (SD)	65.1(19.0)	63.8 (17.4)	0.528
BMI, m/kg2, mean (SD)	26.9 (7.5)	26.3 (6.8)	0.701
HbA1C, mean (SD)	5.3 (0.5)	4.7 (1.7)	0.126
Ethnicity, Malay, n (%)	132 (91.0)	137 (85.6)	0.144
Length of education			0.457
10-11 y, n (%)	29 (20.0)	30 (18.8)	
12 y, n (%)	57 (39.3)	74 (46.3)	
Tertiary degree*, n (%)	59 (40.7)	56 (35.0)	
Employed, n (%)	123 (84.8)	141 (88.1)	0.183
Family history of diabetes, yes, n (%)	70 (48.3)	63 (39.4)	0.090

BMI – body mass index, SD – standard deviation, HbA1C – haemoglobin A1c test

*Tertiary degree means undergraduate degree (or similar) and above.

### Anthropometric outcome measures

During the 33-week intervention period, all women in the intervention group received the required six CHP session dose and downloaded the app, but there was no statistical reduction in WC between the intervention and control groups (**Table 2**). In fact, WC increased by 1.2 cm in the intervention group and 1.0 cm in the control group, although this difference was not statistically significant (*P* = 0.811). Weight increased by 0.8 kg and BMI by 0.3 kg/m^-2^ in the intervention group and 1.6 kg and 0.7 kg/m^-2^ in the control group. These increases were significantly smaller in the intervention compared to the control group (**Table 2**).

**Table 2:** Anthropometric outcomes for all participants: change in waist circumference, weight and BMI in intervention and control (standard of care) groups of the trial*

Outcome variable	Intervention (n = 145)	Control (n = 160)	*P*-values
WC†, cm, mean (SD)	82.6 (13.9)	81.7 (13.9)	0.547
Change in WC‡ (baseline minus endpoint assessment)	-1.19 (6.6)	-1.02 (5.6)	0.811
Weight†, kg, mean (SD)	66.7 (18.8)	65.4 (17.5)	0.528
Change in weight‡ (baseline minus endpoint assessment)	-0.83 (3.4)	-1.6 (2.6)	0.025
BMI†, mean (SD)	27.3 (7.4)	27.0 (7.0)	0.701
Change in BMI‡ (baseline minus endpoint assessment)	-0.33 (1.4)	-0.66 (1.1)	0.019

WC – waist circumference, BMI – body mass index, SD – standard deviation

*BMI according to the WHO Asian definition of underweight/normal weight; overweight and obese.

†Controlled for age-group by bootstrapping.

‡A negative change result indicates a positive gain in the parameter between baseline and endpoint assessment.

A subgroup analysis was conducted on the anthropometric measures according to the categories: underweight/normal weight, overweight, and obese (**Table 3**). No difference was found in the change in WC between the subgroups. However, the weight gain was significantly less in the obese intervention group compared to the obese control subgroup (0.1 kg vs 1.7 kg; *P* = 0.023). In terms of BMI, the obese intervention subgroup showed a slight reduction (0.01) compared to the obese control subgroup, whose BMI increased by 0.7 (*P* = 0.015).

**Table 3:** Subgroup analysis for changes in waist circumference, weight and BMI

Outcome variable*	Intervention (n = 145)	Control (n = 160)	*P*-values
Change in WC†, cm (baseline minus endpoint assessment)			0.907
Under/normal, mean (SD)	-1.8 (5.5)	-0.9 (5.2)	
Overweight, mean (SD)	-0.6 (6.9)	-0.6 (5.8)	
Obese, mean (SD)	-1.2 (7.2)	-1.5 (5.7)	
Change in Weight†, kg (baseline minus endpoint assessment)			0.023
Under/normal, mean (SD)	-1.3 (2.3)	-0.1 (2.0)	
Overweight, mean (SD)	-1.2 (2.7)	- 2.1 (2.7)	
Obese, mean (SD)	- 0.1 (4.5)	-1.7 (2.9)	
Change in BMI†, kg/m^2^ (baseline minus endpoint assessment)			0.015
Under/normal, mean (SD)	-0.5 (0.9)	-0.5 (0.9)	
Overweight, mean (SD)	-0.5 (1.1)	-0.9 (1.1)	
Obese, mean (SD)	0.01 (1.9)	-0.7 (1.2)	

WC – waist circumference, BMI – body mass index, SD – standard deviation

*Data were analysed by an ANOVA-test and presented as mean differences.

†A negative change result indicates a positive gain in the parameter between baseline and endpoint assessment.

### Secondary outcomes

Secondary outcomes were monitored in several domains, including metabolic outcomes (**Table 4**), dietary intake (**Table 5**), physical activity (**Table 6**) and depression, anxiety, and stress levels (**Table 7**).

**Table 4:** Comparison of metabolic outcomes between the intervention and control groups

Outcome variables*	Intervention (n = 145)	Control (n = 160)	*P*-values
BP (systolic), mean (SD)	107.6 (14.0)	104.7 (10.6)	0.031
BP (diastolic), mean (SD)	72.3 (10.5)	70.9 (9.1)	0.230
Elevated BP, n (%)	11 (7.6)	5 (3.1)	0.081
HbA1C, mean (SD)	5.3 (0.6)	5.2 (0.5)	0.093
>6.5mmol/l, n (%)	2 (1.4)	0	0.133
Total cholesterol, mean (SD)	4.86 (0.9)	4.79 (0.9)	0.527
>5.2mmol/l, n (%)	46 (32.2)	44 (27.7)	0.394
HDL, mean (SD)	1.45 (0.4)	1.43 (0.4)	0.632
<1.2mmol/l, n (%)	34 (23.8%)	45 (28.3%)	0.372
Triglycerides, mean (SD)	0.90 (0.5)	0.85 (0.4)	0.364
>1.7mmol/l, n (%)	11 (7.7)	6 (3.8)	0.140

BP – blood pressure, SD – standard deviation, HBA1c – haemoglobin A1c test, HDL – high-density lipoprotein

*Data were analysed by the null-hypothesis that there would be no difference between the intervention and control group; all variables were controlled for age-group by bootstrapping.

**Table 5:** Post-intervention outcome of dietary intake of selected food items post-intervention measured by the Food Frequency Questionnaire (FFQ) in intervention and control groups of the trial

Food items	Intervention (n = 145)	Control (n = 160)	*P*-values
Vegetable intake per week, mean (SD)	9.4 (7.8)	8.7 (6.1)	0.458
Fruit intake per week, mean (SD)	5.8 (4.8)	5.1 (4.2)	0.199
Rice, Portion size, n (%)			0.001
¼ plate	38 (26.2)	23 (14.4)	
½ plate	66 (45.5)	57 (35.6)	
¾ plate	38 (26.2)	70 (43.8)	
1 plate	3 (2.1)	10 (6.3)	
Noodles, Portion size, n (%)			0.333
¼ plate	35 (25.7)	31 (20.8)	
½ plate	70 (51.5)	70 (47.0)	
¾ plate	30 (22.1)	47 (31.5)	
1 plate	1 (0.7)	1 (0.7)	
Bread, Portion size, n (%)			0.175
1	24 (16.9)	19 (12.0)	
2	91 (64.1)	97 (61.0)	
3	27 (19.0)	43 (27.0)	
Fried Foods, Frequency, n (%)			0.803
Rarely (1-3/mo)	17 (11.7)	15 (9.4)	
Occasionally (1-2/week)	43 (29.7)	40 (25.0)	
Frequently (3-6/week)	65 (44.8)	80 (50.0)	
Daily	20 (13.8)	25 (15.6)	
Fast food, Frequency, n (%)			0.770
Rarely (1-3/mo)	105 (75.5)	110 (71.9)	
Occasionally (1-2/week)	28 (20.1)	36 (23.5)	
Frequently (3-6/week)	6 (4.3)	7 (4.6)	
Daily	0	0	
Carbonated drinks, Frequency, n (%)			0.179
Rarely (1-3/mo)	88 (80.0)	93 (69.9)	
Occasionally (1-2/week)	17 (15.5)	33 (24.8)	
Frequently (3-6/week)	5 (4.5)	7 (5.3)	
Daily	0	0	
Pastries, Frequency, n (%)			0.491
Rarely (1-3/mo)	87 (64.6)	81 (57.0)	
Occasionally (1-2/week)	36 (26.7)	46 (32.4)	
Frequently (3-6/week)	12 (8.9)	14 (9.9)	
Daily	0	1 (0.7)	
Sweet local delicacies (‘kuih’), Frequency, n (%)			0.336
Rarely (1-3/mo)	21 (14.9)	33 (21.3)	
Occasionally (1-2/week)	57 (40.4)	50 (32.3)	
Frequently (3-6/week)	53 (37.6)	62 (40.0)	
Daily	10 (7.3)	10 (6.5)	

SD – standard deviation

**Table 6:** Post-intervention physical activity outcomes measured by IPAQ in intervention and control groups of the trial

Item	Intervention (n = 145)	Control (n = 160)	*P*-values
Vigorous* job related physical activity, mins/week, mean (SD)	259.9 (389.7)	153.8 (280.2)	0.032
Moderate† job-related physical activity, mins/week, mean (SD)	749.0 (822.0)	550.0 (725.4)	0.058
Transport, mins/week, mean (SD)	495.0 (983.5)	480.0 (794.7)	0.908
Vigorous* leisure physical activity, mins/week, mean (SD)	120.5 (143.6)	138.5 (164.7)	0.417
Moderate† leisure physical activity, mins/week, mean (SD)	271.8 (463.2)	328.6 (607.0)	0.434
Sitting‡, mins/week, mean (SD)	273.9 (225.0)	321.9 (270.8)	0.133

SD – standard deviation

*Activities that take hard physical effort and make you breathe much harder than normal.

†Activities that take moderate physical effort and make you breathe somewhat harder than normal.

‡Time spent sitting at a desk, visiting friends, reading, or sitting or lying down to watch television.

**Table 7:** Post-intervention outcome measurements on depression, anxiety and stress measured by DASS-21 in the trial’s intervention and control groups

Item	Intervention (n = 145)	Control (n = 160)	*P*-values
Depression, n (%)			0.595
Normal	114 (78.6)	124 (77.5)	
Moderate	29 (20.0)	31 (19.4)	
Severe	2 (1.4)	5 (3.1)	
Anxiety, n (%)			0.635
Normal	77 (53.1)	81 (50.6)	
Moderate	50 (34.5)	53 (33.1)	
Severe	18 (12.4)	26 (16.3)	
Stress, n (%)			0.190
Normal	119 (82.1)	124 (77.5)	
Moderate	23 (15.9)	26 (16.3)	
Severe	3 (2.1)	10 (6.3)	

For dietary intake, the only difference was that 71.7% in the intervention group took half servings or less of rice, as compared to 50% in the control group (*P* = 0.001) (**Table 5**). Furthermore, there was a significant difference in job-related physical activity between the intervention and control groups; 259.9 Metabolic Equivalent for Task (MET)/week vs 153.8 MET/week of vigorous activities (*P* = 0.03) and 749.0 MET/week vs 550.0 MET/week of moderate activities (*P* = 0.058) (**Table 6**).

## DISCUSSION

To the best of our knowledge, Jom Mama is one of the few complex pre-conception interventions that attempted to change health in young adults in a middle-income country. The combination of administering the trial through the health system, with support from academia and the private sector, offered a unique opportunity to capitalise on the outcomes by taking Jom Mama to scale, if appropriate. Furthermore, the trial was prepared in collaboration with end-users, the local government, and the health system managers, based on the provision of resources necessary for its administration. It was developed systematically, by mapping and including relevant preparatory studies. The consort diagram (**Figure 1**) shows a sufficient number of study participants were recruited, so the trial remained adequately powered.

The baseline data for the trial showed that this sample of young adults recruited in the Seremban region had a high prevalence of overweight and obesity (**Table 1**), as is the case for Malaysia nationally [2]. They were also relatively physically inactive, with most activity occurring in the workplace rather than during leisure (**Table 6**).

Over eight months, the Jom Mama intervention trial did not result in differences in change in WC between the intervention vs the control group. There were, however, statistically significant, albeit small differences in weight gain and BMI, where the intervention group had smaller increases than the control group. Subgroup analysis showed that this effect was primarily present among obese women in the cohort. There were no significant effects on the other anthropometric, cardiovascular, metabolic, behavioural, and mental health secondary outcomes. This suggests that, while the intervention had only a small effect on the population’s overall weight, the intervention was beneficial for those women who were already obese.

While the importance of intervening in the pre-conception period to reduce NCD risk in both women and their offspring is widely recognised [15], few interventions were tested in this period rather than in pregnancy [16]. This is partly due to the definition of the pre-conception period, which offers different opportunities depending on the degree of intent to conceive [17].

One limitation of our study comes from the challenge of implementing nutritional and other relevant clinical guidelines in the population, especially in a low resource setting. Similar challenges were faced in other studies [18,19]. The intervention was also delivered to young couples who deemed themselves as healthy individuals without a need for encounters with health care services except for acute episodes, which may have resulted in a lower level of completion of intervention activities. This trial’s strength is the systematic development of the intervention [8-11], staff receiving appropriate training and subsequent support, and the development of a dedicated e-health app. A concurrent process evaluation allowed lessons to be learned about the trial’s potential limitations, including aspects of BCC and e-health implementation, which may have influenced the trial’s outcomes [20]. Furthermore, important lessons were learned about the challenges of recruitment and attrition, which allowed changes to be made, ensuring an adequate sample size. Despite considerable effort, using publicity and numerous promotional materials and exploring several recruitment sites (including community health clinics and marriage registry offices), a low percentage of couples approached agreed to participate in the trial. It is clearly challenging to engage this group and ensure active participation to change behaviour. This finding has important public health implications, discussed in depth in the accompanying process evaluation paper [20].

Despite considerable efforts to train and support the CHPs in delivering the intervention, many did not feel comfortable doing so [20]. This may be due to the BCC principles being antithetical to their training as transmission-focused rather than interaction-focused information communicators. Furthermore, although significant resources were invested in developing the Jom app, our analysis showed that it was not widely accessed [20].

Though the effect on the obese subgroup of women offers hope that an approach similar to Jom Mama might have greater beneficial health effects if amplified in various ways, the scale and the cost of the intervention make this unlikely, at least in its’ present form. Further discussion of the public health conclusions is presented in an accompanying paper [20].

The establishment of a novel private-public partnership, comprising the Malaysian Ministry of Health, academic researchers from several universities, and a private sector funding partner with an interest in metabolic health, was found to be essential in taking on a task of this magnitude. The rationale for pre-conception interventions is strong, so more research is needed in different parts of the world and with different health care systems. However, as the Jom Mama intervention shows, the magnitude of this undertaking is considerable.

## CONCLUSIONS

From a life-course perspective, there is a strong rationale for interventions to improve health among young people for themselves now and in the future, and for their future children. In a Malaysian context, the high levels of overweightness and obesity and low levels of physical activity in young married adults, along with other priorities at this time in their lives, make significant lifestyle and health changes difficult. A successful intervention will depend on striking the balance between sufficient intensity to alter metabolic risk over a short time period and designing it in such a way that it is compatible with the target population’s daily life. Despite the use of local community health workers, a dedicated app, and considerable effort to sustain follow-up, the modest effects of the Jom Mama intervention, primarily on reducing ongoing weight gain in already obese women, make it clear that this is a very challenging problem. The findings of the Jom Mama trial and the accompanying process evaluation [20] provide some guidance to inform future public health policies and health promotion programmes.
